# The complete chloroplast genome sequence of *Vitis davidii* Foex strain ‘SJTU003’

**DOI:** 10.1080/23802359.2019.1673234

**Published:** 2019-10-04

**Authors:** Quanyou Tian, Peining Fu, Wei Wu, Rongfang Li, Lyubka Koleva, Jiang Lu, Shiren Song

**Affiliations:** a Center for Viticulture and Enology, School of Agriculture and Biology, Shanghai Jiao Tong University, Shanghai, China;; b Department of Plant Physiology and Biochemistry, Agricultural University, Plovdiv, Bulgaria

**Keywords:** *Vitis davidii* Foex, Illumina sequencing, chloroplast genome, phylogenetic analysis

## Abstract

In the present study, the complete chloroplast genome of *Vitis davidii* Foex strain ‘SJTU003’ was assembled and subjected to phylogenetic analysis. This chloroplast genome of ‘SJTU003’ was 161,335 bp in length, including two inverted repeat regions (IRa and IRb) that were separated by a large single-copy region (89,570 bp) and a small single-copy region (19,059 bp). The genome contained 133 genes, including 88 protein-coding genes, 37 tRNA genes, and 8 rRNA genes. Phylogenetic analysis indicated that *V. davidii* is most closely related to *Vitis flexuosa* and *Vitis amurensis*.

*Vitis davidii* Foex, which is one of the native wild grape species found in south central China, has the potential to be used for making wine and juice (Yi et al. 2019). To make better use of *V. davidii*, the complete chloroplast genome of *V. davidii* strain ‘SJTU003’ was assembled (GenBank: MN181471) and subjected to phylogenetic analysis.

Fresh ‘SJTU003’ leaves were sampled from the Research Vineyard of Grape Germplasm and Breeding at Shanghai Jiao Tong University, 800 Dongchuan Road, Minhang District, Shanghai, China (121°26′E; 31°02′N), and total genomic DNA was extracted using the CTAB method (Fu et al. 2019). The DNA was stored at −80 °C at the Center of Viticulture and Enology of Shanghai Jiao Tong University and then sequenced using the HiSeq X pair-end 150 sequencing platform (Illumina, San Diego, CA). A total of 3.79 Gb clean data were obtained, and chloroplast genome was assembled using SOAPdenovo v2.04 (Luo et al. 2012) with the chloroplast genome sequence of *Vitis vinifera* (DQ424856) as a reference. The DOGMA package (Wyman et al. 2004) and BLAST 2.6.0+ (ftp.ncbi.nih.gov/blast/) were used to predict and annotate genes, respectively.

This chloroplast genome of ‘SJTU003’ was 161,335 bp in length, including two inverted repeat (IR) regions (IRa and IRb) that were separated by a large single-copy (LSC) region (89,570 bp) and a small single-copy (SSC) region (19,059 bp). The GC content of the genome was 37.4%, whereas that of the LSC and SSC regions were 35 and 32% in SSC, and that of the IR regions was 43%. The genome contained 133 genes, including 88 protein-coding genes, 37 tRNA genes, and 8 rRNA genes. Among these genes, 19 contained one intron (*tRNA-Lys*, *rps16*, *tRNA-Gly*, *atpF*, *rpoC1*, *tRNA-Leu*, *tRNA-Val*, *petB*, *petD*, *rpl16*, two *rpl2*, two *ndhB*, two *tRNA-Ile*, two *tRNA-Ala*, and *ndhA*), and 4 contained two introns (*ycf3*, *clpP,* and two *rps12*).

To investigate the phylogenetic position of *V. davidii* within the Vitaceae, the chloroplast genome sequence of ‘SJTU003’ and those of 16 other *Vitis* species were aligned using MAFFT version 7 (Katoh and Standley 2013). A neighbour-joining phylogenetic tree was constructed using MEGA X (Kumar et al. 2018; Figure 1). The phylogenetic position of different *V. davidii* Foex strains had diversity, and *V. davidii* was most closely related to *Vitis flexuosa* and *Vitis amurensis*.

**Figure 1.**
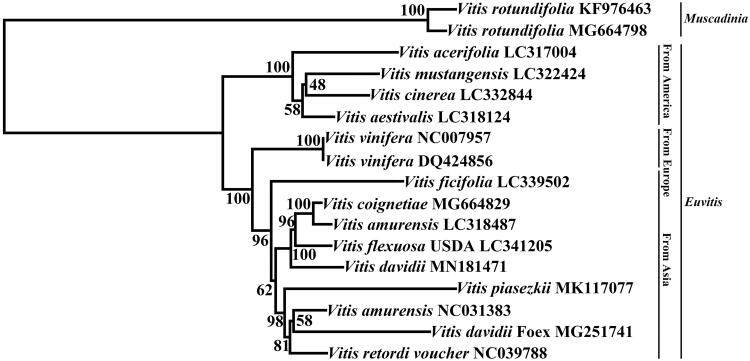
The phylogenetic relationship of 17 species within the *Vitis* species based on neighbour-joining (NJ) analysis of chloroplast genomes. The bootstrap values are based on 1000 replicates and are shown next to the nodes.
